# A New *SLC10A7* Homozygous Missense Mutation Responsible for a Milder Phenotype of Skeletal Dysplasia With Amelogenesis Imperfecta

**DOI:** 10.3389/fgene.2019.00504

**Published:** 2019-05-28

**Authors:** Virginie Laugel-Haushalter, Séverine Bär, Elise Schaefer, Corinne Stoetzel, Véronique Geoffroy, Yves Alembik, Naji Kharouf, Mathilde Huckert, Pauline Hamm, Joseph Hemmerlé, Marie-Cécile Manière, Sylvie Friant, Hélène Dollfus, Agnès Bloch-Zupan

**Affiliations:** ^1^ Laboratoire de Génétique Médicale, UMR_S INSERM U1112, Faculté de Médecine, FMTS, Institut Génétique Médicale d’Alsace (IGMA), Université de Strasbourg, Strasbourg, France; ^2^ Laboratoire de Génétique Moléculaire, Génomique, Microbiologie (GMGM), UMR7156, Centre National de Recherche Scientifique (CNRS), Université de Strasbourg, Strasbourg, France; ^3^ Service de Génétique Médicale, Hôpitaux Universitaires de Strasbourg, IGMA, Strasbourg, France; ^4^ Faculté de Chirurgie Dentaire, Université de Strasbourg, Strasbourg, France; ^5^ Laboratoire de Biomatériaux et Bioingénierie, Inserm UMR_S 1121, Strasbourg, France; ^6^ Pôle de Médecine et Chirurgie Bucco-dentaires, Hôpital Civil, Centre de référence des maladies rares orales et dentaires, O-Rares, Filière Santé Maladies rares TETE COU, European Reference Network ERN CRANIO, Hôpitaux Universitaires de Strasbourg (HUS), Strasbourg, France; ^7^ Centre de Référence pour les affections rares en génétique ophtalmologique, CARGO, Filière SENSGENE, Hôpitaux Universitaires de Strasbourg, Strasbourg, France; ^8^ Institut de Génétique et de Biologie Moléculaire et Cellulaire (IGBMC), INSERM U1258, CNRS-UMR7104, Université de Strasbourg, Illkirch-Graffenstaden, France; ^9^ Eastman Dental Institute, University College London, London, United Kingdom

**Keywords:** skeletal dysplasia, amelogenesis imperfecta, NGS (next generation sequencing), human, rare diseases

## Abstract

Amelogenesis imperfecta (AI) is a heterogeneous group of rare inherited diseases presenting with enamel defects. More than 30 genes have been reported to be involved in syndromic or non-syndromic AI and new genes are continuously discovered (Smith et al., 2017). Whole-exome sequencing was performed in a consanguineous family. The affected daughter presented with intra-uterine and postnatal growth retardation, skeletal dysplasia, macrocephaly, blue sclerae, and hypoplastic AI. We identified a homozygous missense mutation in exon 11 of *SLC10A7* (NM_001300842.2: c.908C>T; p.Pro303Leu) segregating with the disease phenotype. We found that *Slc10a7* transcripts were expressed in the epithelium of the developing mouse tooth, bones undergoing ossification, and in vertebrae. Our results revealed that SLC10A7 is overexpressed in patient fibroblasts. Patient cells display altered intracellular calcium localization suggesting that SLC10A7 regulates calcium trafficking. Mutations in this gene were previously reported to cause a similar syndromic phenotype, but with more severe skeletal defects (Ashikov et al., 2018;Dubail et al., 2018). Therefore, phenotypes resulting from a mutation in *SLC10A7* can vary in severity. However, AI is the key feature indicative of *SLC10A7* mutations in patients with skeletal dysplasia. Identifying this important phenotype will improve clinical diagnosis and patient management.

## Introduction

Skeletal dysplasias are a heterogeneous group of diseases affecting bone and cartilage formation resulting in short stature. These diseases are associated with shorter long bones and abnormal shape and/or size of the skeleton, spine, and head and, eventually other anomalies (including neurological, cardiac, and respiratory defects). Distinguishing individual pathologies in this wide group of diseases is improving thanks to advances in genomic technologies and genetic analyses (Alanay and Lachman, 2011).

Enamel and bone formation share a common mineralization process involving the precipitation of inorganic hydroxyapatite nanocrystals within organic matrices to form biological structures (Abou Neel et al., 2016). When fundamental mineralization processes are disrupted, skeletal dysplasia-associated syndromes can include enamel alterations.

Amelogenesis imperfecta (AI) is a heterogeneous group of rare inherited diseases presenting with anomalies in dental enamel formation. More than 30 genes are known to be involved in AI, and new genes are continuously being discovered (Smith et al., 2017).

Here, we report the case of a patient with a mutation in *SLC10A7,* a potential calcium transporter, presenting with skeletal dysplasia associated with AI. We identified the first *SLC10A7* missense mutation occurring at the end of the protein (p.Pro303Leu) leading to milder skeletal anomalies compared to previously described patients with mutations in this gene (Ashikov et al., 2018; Dubail et al., 2018).

## Materials and Methods

### Patient

**Figure 1:**
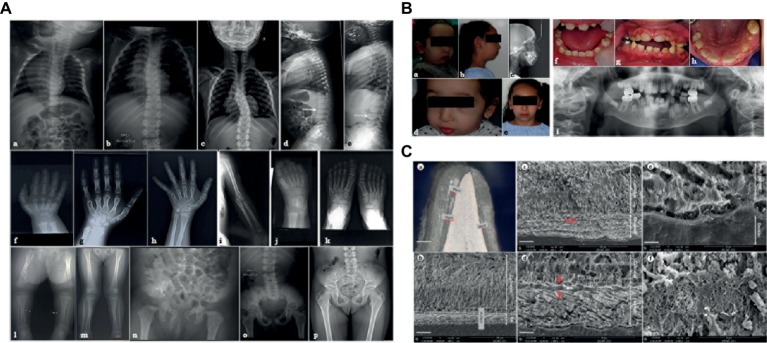
**(A)** X-ray images of the skeletal anomalies at 3 months old **(a,d,f,j,l,n)**, 4 years old **(b,e,g,i,k,m,o)** and 9 years old **(c,h,p)**. Radiographs of the vertebral column showed the progressive scoliosis **(a–c)** and the ballooning of the vertebrae **(d,e)**. Radiographs of hands and feet **(f–h,j,k)** displayed a progressive carpal and tarsal ossification. The patient also presented with short long bones **(i,l,m)**, a genu valgus **(l)**, and a horizontal acetabulum **(n–p)**. **(B)** Clinical and radiological images of the facial and dental anomalies at 2 years old **(a,d,f)** and 9 years old **(b,c,e,g–i)**. AI is visible in the clinical intraoral photographs affecting both the primary **(f)** and permanent dentition **(g,h)**. The panoramic radiograph showed no radio-opacity contrast between dentine and enamel **(i)**. **(C)** Optical numeric microscope and scanning electronic microscopy (SEM) of primary teeth. Numeric optical view of a sagittal section of a primary tooth. Dentine appears brighter than enamel. Lower central primary incisor **(a)**. SEM micrograph of the thin enamel layer covering the vestibular side of the tooth. A thick layer of calcified calculus is present **(b)**. Higher magnification of the thin enamel layer. Pseudo-prismatic structures are evident (arrows) **(c)**. Micrograph of the thin enamel showing an outer aprismatic layer (between red arrows) exhibiting incremental lines (white arrow) **(d)**. Enamel-dentinal junction showing typical scallops and unusual non-mineralized collagen fibers at the interface **(e)**. Higher magnification of the calculus material capping the tooth. Many entangled calcified filamentous bacteria are observed **(f)**.

The patient was examined at the Centre de Référence des Maladies Rares orales et dentaires (O-Rares), Strasbourg, France and at the Centre de Compétence des Maladies Osseuses Constitutionnelles (OSCAR) in Strasbourg. The oral phenotype was documented using the D[4]/phenodent registry protocol^1^. This clinical study is registered at https://clinicaltrials.gov: NCT01746121/NCT02397824 and with the French Ministry of Higher Education and Research Bioethics Commission as a biological collection “Orodental Manifestations of Rare Diseases” DC-2012-1677/DC-2012-1002 and was acknowledged by the person protection committee. The parents gave written informed consents for the genetic analyses performed on the salivary samples both for them and their children in accordance with the Declaration of Helsinki. They also gave written consent for the publication of this case report and the images of their daughter presented in Figure 1.

### Electron Microscopy

Teeth were washed, stored in 70% ethanol at 4°C, embedded in Epon 812 (Euromedex, France), sectioned, and polished. Sections were then etched with 20% (w/w) citric acid, dehydrated, and analyzed by using an optical numeric microscope (KEYENCE, Japan) and a Quanta 250 FEG scanning electron microscope (SEM) (FEI Company, the Netherlands). Specimens also underwent chemical analysis (see Supplementary Methods).

### Whole-Exome Sequencing

Whole-exome sequencing (WES) was performed on the affected patient (II.4) and her parents (I.1 and I.2) by Integragen (Evry, France, 2014). Exons were captured using SureSelect Human All Exon Kits (Agilent, France) with the company’s probe library (Agilent Human All Exon v5 + UTR 75 Mb Kit) and sequenced with an Illumina HISEQ2000 (Illumina, USA) as paired-end 75 bp reads, resulting in an average coverage of 80X. The sequence reads were then aligned to the reference sequence of the human genome (GRCh37) (see Supplementary Methods).

### Bioinformatics Analysis

Annotation and ranking of SNV/indel were performed by VaRank (Geoffroy et al., 2015) in combination with the Alamut Batch software (Interactive Biosoftware, France) (see Supplementary Methods).

### Sample Collection and Sanger Sequencing and Segregation

Saliva samples from the affected daughter, her unaffected parents, and siblings were collected (I.1, I.2, II.4, II.1, II.2, and II.3). The amplification of the region of interest (see Supplementary Table S1 for primers sequences) was performed on genomic DNA template followed by a bidirectional Sanger sequencing (see Supplementary Methods).

### Multiple Protein Sequences Alignment

The SLC10A7 human last transmembrane domain (TM10) was aligned with the SLC10A7 sequence of other species using Uniprot website^2^. The data were then imported and visualized with Jalview^3^ and colored according to the “ClustalX” coloring scheme.

### *In situ* Hybridization

Mouse embryos embedding and sectioning, probe synthesis and *in situ* hybridization were performed as previously described in Laugel-Haushalter et al., 2012. DIG-labeled antisense riboprobe was transcribed *in vitro* with SP6 RNA polymerase (see Supplementary Figure S1 for template sequence). The experiments were realized in accordance with the European Community Council Directive (86/609/EEC).

### Western Blot Analysis

Patient and control primary fibroblasts were grown in DMEM (2 mM glutamine, 10% FCS), collected, and lysed in Ripa buffer containing protease cocktail inhibitor (Roche 06538282001). Proteins obtained were used for Western blot, where SLC10A7 was detected with a specific monoclonal antibody (Novus NBP1-59875), followed by secondary HRP-coupled antibody hybridization (NA934V, GE Healthcare). Detection by ECL using the ChemiDoc™ Touch (BioRad) imaging system was performed. Quantification was performed using the ImageLab software (BioRad). SLC10A7 was quantified relative to the total amount of protein per lane revealed by TCE (T54801 Sigma) UV-labeling of the tryptophan residues, as shown on the stain-free loading control.

### Calcium Localization

The control and patient fibroblasts were grown to 75% confluence in six-well plates with sterile coverslips. Cells were then incubated with 4 μM Fluo4-AM (Thermo Fisher Scientific, #F14201) in DMEM medium (2 mM glutamate, 10% FBS) for 15 or 30 min at RT, quickly rinsed with fresh medium without Fluo4, and rinsed a second time for 15 or 30 min at RT. Cells were mounted directly in PBS and observed on a Zeiss Axio Observer D1 fluorescent microscope using a 40X objective.

## Results

### Patient Phenotype

The investigated patient, a girl, is the consanguineous fourth child of an Algerian couple with no reported personal or family medical history. She presented with intra-uterine growth retardation and short femurs detected during the third trimester of pregnancy. The child was born at term with a birth height of 42 cm (<<third percentile), a birth weight of 2,890 g (10th percentile), and a head circumference of 32.5 cm (10th percentile). Rhizomelia and brachydactyly of hands and feet were noticed at birth.

Her growth was regular at −3SD for height, −2.5SD for weight, and −1SD for head circumference. Clinical examination revealed joint laxity without dislocations, articular limitations or pain, and a progressive scoliosis. By age 7, the child measured 106 cm (−3SD), weighted 18 kg (−2.5SD), and had a head circumference of 51 cm (0SD).

Radiographs were undertaken at 3 months (Figures 1Aa,Ad,Af,Aj,Al,An), at 4 years (Figures 1Ab,Ae,Ag,Ai,Ak,Am,Ao), and at 9 years (Figures 1Ac,Ah,Ap). Advanced carpal ossification, brachymetacarpia (Figures 1Af–Ah.), and short long bones (Figures 1Ai,Al,Am) were noticed at birth. Mesomelia grew more evident with age (Figures 1Af–Ah). Tarsal bone ossification was also advanced (Figures 1Aj,Ak). Vertebral bodies were initially considered as normal, although the latest radiographs showed abnormal expansions or ballooning (Figures 1Ad,Ae). Spinal hyperlordosis and scoliosis developed over time (Figures 1Aa–Ac). Horizontal acetabulum, large femoral necks, large iliac wings, and large clavicles were also noticed (Figures 1An–Ap). Neither metaphyseal nor epiphyseal anomalies were observed (Figures 1Al,Am). Different diagnoses of constitutional bones diseases such as achondroplasia and hypochondroplasia suggested Silver-Russell syndrome and diastrophic dwarfism, but molecular analyses ruled out these syndromes. Array-CGH was also normal (data not shown). The child had normal psychomotor development, although some learning difficulties were recently noticed.

The patient also presented with facial dysmorphisms, including microretrognathia, short neck, short nose, flat face, and blue sclerae (Figures 1Ba,Bb,Bd,Be). She had a narrow pharyngeal tract, a hypodivergent profile with protruding incisors, and biproalveoly to compensate (Figure 1Bc) a lingual dysfunction and lip inocclusion.

The child had smaller teeth, incisor infraclusion, and severe hypoplastic/hypomineralized amelogenesis imperfecta on both the primary and permanent dentitions with colored and softer enamel (Figure 1B). On radiographs, there was no radio-opacity contrast between hypomineralized enamel and dentine (Figure 1Bi).

Initial ENT and ophthalmologic examinations were normal. Mild hypermetropia and astigmatism were then noticed in the first years of life.

### Enamel Shows Quantitative and Qualitative Defects

Optical microscopy assessments of sagittal sections of a primary naturally exfoliated tooth revealed severe enamel hypoplasia (Figure 1Ca). The maximum thickness of enamel was observed on the vestibular side of the tooth and reached 80 μm (around 700 μm in a similar control tooth), and the entire tooth was capped by dental calculus. Figure 1Cb shows the large thickness of the calculus capping compared to the narrow enamel layer. In SEM, the thin enamel exhibited wide pseudo-rod patterns (Figure 1Cc). Moreover, electron microscopy disclosed a very thin outer layer of aprismatic enamel with incremental lines parallel to the surface (Figure 1Cd). At the scalloped enamel-dentinal junction, a non-mineralized interphase of collagen fibrils was evident (Figure 1Ce). At this higher magnification, the individual enamel crystals could be observed. Microscopy observations of the calculus capping clearly showed calcified bacterial structures (Figure 1Cf). When analyzed by energy dispersive X-ray spectroscopy (EDX), the calculus material had a Ca/P ratio of 1.56 ± 0.04 (*n* = 12) (Supplementary Figure S2A). Comparatively, EDX measurements were, respectively, 1.74 ± 0.068 (*n* = 15) and 1.64 ± 0.062 (*n* = 15) for enamel and dentine tissues (Supplementary Figure S2B). Although both spectra looked similar, the carbon content was higher in dental calculus, i.e., C = 15.4 ± 0.85 (wt.%) and C = 5.13 ± 0.93 (wt.%) for enamel (Supplementary Figure S2).

### Mutation in *SLC10A7* Associated With Syndromic Amelogenesis Imperfecta

**Figure 2:**
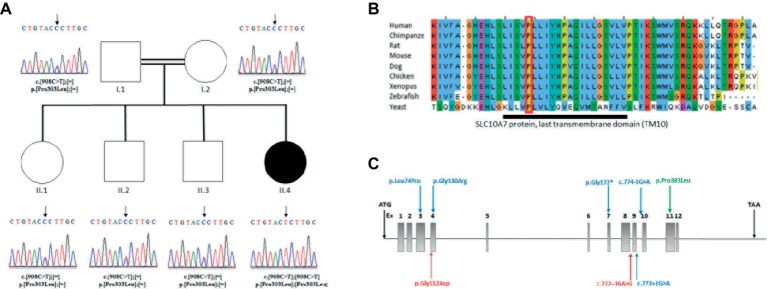
**(A)** Analysis of the *SLC10A7* mutation: Pedigree of the AI patient and DNA sequencing chromatograms of the whole family. Parents and other children were heterozygous and the patient was homozygous for the mutation. An arrow points to the mutation. **(B)** Multiple sequence alignment of SLC10A7 last transmembrane domain (TM10). The largely conserved sequence of the proteins last transmembrane domain is represented by the dark bar. The amino-acid affected by the missense mutation in the patient (red square) is conserved from human to yeast. **(C)** Human *SLC10A7* mutations in the literature. The *SLC10A7* gene contains 12 exons. The mutations described in Dubail et al. (2018) are represented by blue arrows and those reported in Ashikov et al., 2018 by red arrows. Our mutation is the only mutation located at the end of the gene (exon 11) and is represented by a green arrow.

Whole-exome sequencing was performed on the index case and her parents. By using stringent criteria, identifying variants consistent with autosomal recessive disease inheritance (Supplementary Table S2), by manual curation (Supplementary Table S3), and by Sanger sequencing (Supplementary Figure S3, Figure 2A), we validated the homozygous mutation in exon 11 of *SLC10A7,* a gene involved in a novel type of skeletal dysplasia associated with short stature, AI, and scoliosis (SSASKS, #OMIM618363) (Ashikov et al., 2018; Dubail et al., 2018). The mutation leading to an amino acid change from proline to leucine in exon 11 at position 303 (NM_001300842.2:c.908C>T, p.Pro303Leu) was absent from databases (1,000 genomes, GnomAD).

The mutation affected the last transmembrane domain (TM10) of the SLC10A7 protein. The alignment of this domain sequence with sequences of other species showed that the sequence was largely conserved between species, with the mutated proline (Pro303) being highly conserved (Figure 2B). The mutation was also predicted to be disease causing by SIFT (Vaser et al., 2016) and deleterious by Polyphen2 (Polymorphism Phenotyping v2) (PPH2) (Adzhubei et al., 2010). Collectively, these findings suggest an important function for this amino acid.

### Expression Pattern of *SLC10A7* in Developing Mouse Bone and Tooth

The expression of *Slc10a7* at E14.5 mouse tooth cap stage had been reported in our previous transcriptomic study showing that the gene was expressed at similar levels in both molars and incisors (Supplementary Table S4; Laugel-Haushalter et al., 2012).

**Figure 3:**
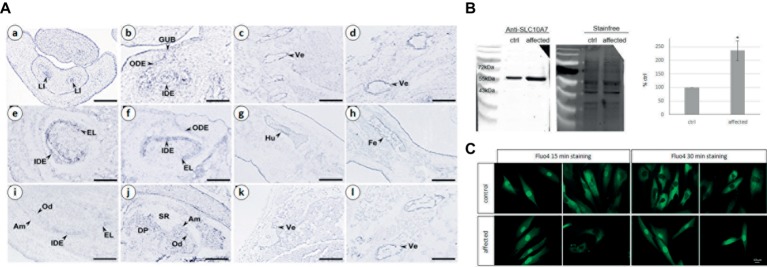
**(A)** Analysis of mouse *Slc10a7* transcripts distribution by *in situ* hybridization. Sections illustrate *Slc10a7* expression in the developing bone, vertebrae, molars, and incisors. Developmental stages and section planes are: E14.5 frontal **(a,b)**, E16.5 sagittal **(c–h)**; E18.5 sagittal **(i–l)** sections. Am, ameloblasts; DP, dental papila; EL, epithelial loop; GUB, gubernaculum; IDE, inner dental epithelium; LI, lower incisor; Od, odontoblasts; ODE, outer dental epithelium; SR, stellate reticulum; Ve, vertebrae. Scale bars: 10 μm **(b)**; 40 μm **(a,c,g,h)**; 30 μm **(d,l)**; 60 μm **(i,j)**; 80 μm **(e,f)**; 150 μm **(k)**. **(B)** SLC10A7 expression in control and patient skin fibroblasts as detected by western blot. The stain-free membrane, displaying the total amount of protein loaded, was used for quantification. The western blot shown corresponds to one significant experiment (*n* = 3). The data plotted were the mean of three independent experiments. Statistical analysis was done with the *t*-test and the *p* was determined *p < 0.05. **(C)** Calcium localization in control and patient fibroblasts. The cells were stained for 15 min or 30 min with 4 μm of Fluo4, then washed for 15 or 30 min, respectively and observed by fluorescent microscopy. Images were taken with a 400× magnification, scale bar 10 μm.

To gain insight into *Slc10a7* gene expression during bone and tooth development in mice, we performed an *in situ* hybridization analysis on mouse fetuses. *Slc10a7* positive signals were observed in the epithelial compartment of E14.5 cap stage teeth (Figures 3Aa,Ab). At E16.5, the transcripts were mostly localized in the inner dental epithelium and in the epithelial loop of the bell stage teeth. A discrete expression was also detected in the outer dental epithelium. At E18.5 labeling was observed in the inner dental epithelium of incisors and in ameloblasts and odontoblasts of molars. *Slc10a7* expression was investigated in developing bones, and mRNA transcripts were detected in bones undergoing ossification and vertebrae. We detected an expression not only in vertebrae at E16.5 (Figures 3Ac,Ad) and E18.5 (Figures 3Ak,Al) but also in the E16.5 humerus (Figure 3Ag) and femur (Figure 3Ah).

### SLC10A7 Is Overexpressed in Patient Fibroblasts

The SLC10A7 protein was identified by western blot analysis in protein extracts from patient and unaffected control primary skin fibroblasts (Figure 3B). Quantifications were done in three independent experiments using total protein extract (as revealed by the stain-free labeling) as loading control. The level of SLC10A7 protein was approximately two times higher in the patient’s cells compared to the unaffected control individual cells (Figure 3B). The results shown here were further validated using two different SLC10A7 commercial antibodies (data not shown).

### Abnormal Distribution of Cellular Calcium in Patient Fibroblasts

As the SLC10A7 yeast *Saccharomyces cerevisiae* homologue (termed RCH1) is involved in the regulation of a calcium transporter (Zhao et al., 2016), this prompted us to investigate the distribution of calcium in patient’s and control fibroblasts. Calcium labeling with a Fluo4 probe showed no difference in localization after 15 min of staining (Figure 3C). However, after 30 min, while the probe was mainly localized within the cytoplasm in the control cells, it was mostly retained within the nucleus in the patient’s fibroblasts. This suggests that SLC10A7 could be involved in calcium transport and that the mutation of the patient resulting in an increased level of protein interferes with this function (Figure 3C).

## Discussion

### Human Mutations

The study by (Dubail et al., 2018) described six patients with homozygous mutations in the *SLC10A7* gene presenting with SSASKS (#OMIM618363). Two patients had splice mutations in intron 9 c.774-G>A, c.773+1G>A; two had missense mutations c.221T>C and c.388G>A; and two showed the same stop mutation c.514C>T. Ashikov et al., 2018 reported two siblings patients with a *SLC10A7* compound heterozygous mutation c.335G>A and c.722–16A>G and two patients with the same clinical presentation with no *SLC10A7* cDNA-identified mutations. In this paper, we report a homozygous missense mutation in exon 11, the only missense mutation reported to date at the end of this protein. The location near the end of the protein could potentially explain the milder phenotype in our patient (Figure 2C).

The patients described by (Dubail et al., 2018) displayed a more severe phenotype with multiple joint dislocations and had clinical features resembling Desbuquois syndrome (OMIM#251450, #615777 caused by mutations in *CANT1* and *XYLT1* genes), a chondrodysplasia with defects in GAG biosynthesis. The only distinguishable feature between those patients and Desbuquois-like patients was the AI phenotype (Table 1). In the study by Ashikov et al., 2018, patients were believed similar to Desbuquois syndrome, sharing some intellectual disability traits not observed in our patient. However, Ashikov et al., 2018 patients also presented AI, a feature not reported so far in Desbuquois syndrome (Table 1). Comparing our patient to Desbuquois dysplasia patients, growth retardation was less severe (−3SD compare to −4 to −10SD). The patient also did not present with multiple dislocations or characteristic features like accessory ossification centers distal to the second metacarpal, bifid distal phalanx, or delta phalanx of the thumb. However, our patient had a similar facial appearance–round face, microretrognathism, blue sclerae, prominent eyes, and a short neck. Like other reported patients, our proband presented with AI. Since classical Desbuquois syndrome patients do not present this phenotype, AI may be a key symptom to distinguish *SLC10A7*-related disorders from other chondrodysplasias.

**Table 1:** Review of the literature of *SLC10A7* patients and clinical comparison with Desbuquois syndrome.

Clinical features	Reported patients (Dubail et al., 2018)	Reported patients (Ashikov et al., 2018)	Our patient	Desbuquois syndrome
Intra-uterine growth retardation	6/6		+	+
Postnatal growth retardation	6/6	5/5	+ (−3SD)	+ (−4SD to −10SD)
Micrognathia	6/6	2/5	+	+
Congenital multiple dislocations	6/6	–	−	+
Amelogenesis imperfecta	6/6	5/5	+	−
Advanced carpal ossification	6/6		+	+
Scoliosis	6/6	5/5	+	+
Blue sclerae			+	+
Prominent eyes		+	+	+
Flat face			+	+
Short neck		+	+	+
Presence of hand anomalies, namely, accessory ossification center distal to the second metacarpal, bifid distal phalanx, or delta phalanx of the thumb	−		–	+ (type 1)
Brachydactyly		+	+	+
Short long bones with “Swedish key” appearance of the proximal femur	4/6			+
Organ malformations			–	+/−
Mental retardation		4/5	–	+/−

The patients from Dubail et al. (2018) presented with a phenotype close to the Desbuquois syndrome phenotype. Patient from (Ashikov et al., 2018) and our proband did not present joint dislocation. All the patients were affected by a skeletal dysplasia associated with AI.

### Mutant Mice and *SLC10A7* Expression Pattern

*Slc10a7^−/−^* null mice exhibited a dysmorphic face, moderate skeletal dysplasia, ligamentous laxity, a reduced bone mass (Dubail et al., 2018) and they were smaller with shorter limbs (Brommage et al., 2014). In the mouse incisors, the most external layer, the aprismatic enamel layer, was missing, and numerous areas of hypoplasia were observed in the external prismatic enamel layer (Brommage et al., 2014; Dubail et al., 2018). As they mimic many clinical features of the patient phenotype, they provide a good model to study the physio-pathological mechanisms of the syndrome. It is interesting to note that no joint dislocations were observed, suggesting that *Slc10a7^−/−^* mice are mimicking the milder phenotype observed in our patient and in previous studies (Brommage et al., 2014). Here, we also showed that the expression pattern of *Slc10a7* in mice was consistent with the organs affected in all the described patients. Indeed, the gene was expressed in vertebrae, bones undergoing ossification and in the epithelial part of the tooth. Those findings are supporting the fact that even if the phenotype of SSAKS patients can vary in severity and include extra clinical features, short stature, scoliosis, and AI are recurrent features to identify patients with mutation in *SLC10A7*.

### Role of SLC10A7 in Calcium Transport

Here, we show that the level of SLC10A7 protein is two times higher in affected patient skin fibroblasts compared to controls. Moreover, incubation of patient cells with a fluorescent calcium probe (Fluo4) led to its accumulation into the nucleus (cytoplasmic localization observed in control cells). This suggests that SLC10A7 may play a role in calcium homeostasis, as reported for the SLC10A7 yeast homologue Rch1 (Jiang et al., 2012). Indeed, in yeast, Rch1 is expressed in response to increased calcium levels and acts as a negative regulator of calcium uptake by binding to a calcium transporter at the plasma membrane (Jiang et al., 2012). Based on these yeast results, SLC10A7 may also be involved in calcium homeostasis, and the patient missense mutations could alter this function, potentially leading to calcium accumulation in the nucleus of patient fibroblasts and higher expression of SLC10A7 to compensate for this defect.

Previous studies concluded that defects in calcium uptake led to defects in GAG synthesis explaining SLC10A7 phenotypes. This mechanism could also produce skeletal defects. Dental defects, though, could be due to a small misregulation in calcium homeostasis, sufficient to induce enamel defects. Moreover, as discussed in Dubail et al., 2018, some mutations in calcium channels and transporters/exchangers (*STIM1, ORAI1, SLC24A4, SLC24A5, WDR72*) (El-Sayed et al., 2009; Feske, 2010; Duan, 2014; Wang et al., 2014) lead to AI without any associated GAG biosynthesis defects.

### Amelogenesis Imperfecta as a Key Clinical Feature in Delineation of Skeletal Dysplasia

Skeletal dysplasias are a large, diverse group of diseases in which recent progress in genetics has allowed identification of many causative genes. An accurate clinical examination can also provide key diagnostic clues. Here, we report the case of a patient with a skeletal dysplasia associated with AI. Skeletal dysplasia with associated *SLC10A7* mutations varies in severity and clinical features (Ashikov et al., 2018; Dubail et al., 2018) but AI is always present. Indeed, mutation in *SLC10A7* was recently associated to a novel type of skeletal dysplasia with short stature, AI, and scoliosis (#OMIM611459). To date, combined skeletal dysplasia/AI-associated syndromes have been observed in non-lethal Raine syndrome with mutations in *FAM20C* (OMIM#259775) (Elalaoui et al., 2016), brachyolmia with AI caused by mutations in *LTBP3* (Huckert et al., 2015), tricho-dento-osseus syndrome caused by mutations in *DLX3* (OMIM#190320) (Nieminen et al., 2011), and congenital disorder of glycosylation, type IIk caused by mutations in *TMEM165* (OMIM #614727).

As the association of the two clinical features seems to be rare, this suggests that AI is the key factor pointing to a mutation in *SLC10A7* in patients presenting with skeletal dysplasia and scoliosis. Moreover, comparing our patient to those in the literature (Ashikov et al., 2018; Dubail et al., 2018), we noticed that the skeletal phenotype was milder, suggesting that some patients could be difficult to diagnose. Thus, the finding of AI can help improving patient diagnosis.

## Ethics Statement

The oral phenotype was documented using the D[4]/phenodent registry protocol, a Diagnosing Dental Defects Database (see www.phenodent.org, for assessment form), which is approved by CNIL (French National commission for informatics and liberty, number 908416). This clinical study is registered at https://clinicaltrials.gov: NCT01746121 and NCT02397824 and with the MESR (French Ministry of Higher Education and Research) Bioethics Commission as a biological collection “Orodental Manifestations of Rare Diseases” DC-2012-1677 within DC-2012-1002 and was acknowledged by the CPP (person protection committee) Est IV December 11th 2012. The patient and the non-affected family members gave written informed consents in accordance with the Declaration of Helsinki, both for the D[4]/phenodent registry and for genetic analyses performed on the salivary samples included in the biological collection.

## Author Contributions

ES, MH, PH, YA, and M-CM collected the salivary samples and detailed the patients’ phenotype. VL-H, CS, and MH identified the molecular basis of the disease through NGS assays. VL-H, SB, ES, CS, VG, NK, JH, M-CM, SF, HD, and AB-Z analyzed the data and wrote the manuscript. AB-Z designed the study and was involved from conception, fund seeking to drafting, and critical review of the manuscript. All authors therefore contributed to the conception, design, data acquisition, analysis, and interpretation, drafted and critically revised the manuscript. All authors gave final approval and agreed to be accountable for all aspects of the work.

### Conflict of Interest Statement

The authors declare that the research was conducted in the absence of any commercial or financial relationships that could be construed as a potential conflict of interest.

## Footnotes

^1^www.phenodent.org

^2^http://www.uniprot.org

^3^http://www.jalview.org/

## References

[ref1] Abou Neel E. A. Aljabo A. Strange A. Ibrahim S. Coathup M. Young A. M. (2016). Demineralization–remineralization dynamics in teeth and bone. Int. J. Nanomedicine 11, 4743–4763. 10.2147/IJN.S107624 27695330 PMC5034904

[ref2] Adzhubei I. A. Schmidt S. Peshkin L. Ramensky V. E. Gerasimova A. Bork P.. (2010). A method and server for predicting damaging missense mutations. Nat. Methods 7, 248–249. 10.1038/nmeth0410-248, PMID: 20354512 PMC2855889

[ref3] Alanay Y. Lachman R. S. (2011). A review of the principles of radiological assessment of skeletal dysplasias. J. Clin. Res. Pediatr. Endocrinol. 3, 163–178. 10.4274/jcrpe.463, PMID: 22155458 PMC3245489

[ref4] Ashikov A. Abu Bakar N. Wen X.-Y. Niemeijer M. Rodrigues Pinto Osorio G. Brand-Arzamendi K.. (2018). Integrating glycomics and genomics uncovers SLC10A7 as essential factor for bone mineralization by regulating post-Golgi protein transport and glycosylation. Hum. Mol. Genet. 27, 3029–3045. 10.1093/hmg/ddy213, PMID: 29878199

[ref5] Brommage R. Liu J. Hansen G. M. Kirkpatrick L. L. Potter D. G. Sands A. T.. (2014). High-throughput screening of mouse gene knockouts identifies established and novel skeletal phenotypes. Bone Res. 2:14034. 10.1038/boneres.2014.34, PMID: 26273529 PMC4472125

[ref6] Duan X. (2014). Ion channels, channelopathies, and tooth formation. J. Dent. Res. 93, 117–125. 10.1177/0022034513507066 24076519

[ref7] Dubail J. Huber C. Chantepie S. Sonntag S. Tüysüz B. Mihci E.. (2018). SLC10A7 mutations cause a skeletal dysplasia with amelogenesis imperfecta mediated by GAG biosynthesis defects. Nat. Commun. 9:3087. 10.1038/s41467-018-05191-8, PMID: 30082715 PMC6078967

[ref8] Elalaoui S. C. Al-Sheqaih N. Ratbi I. Urquhart J. E. O’Sullivan J. Bhaskar S.. (2016). Non lethal Raine syndrome and differential diagnosis. Eur. J. Med. Genet. 59, 577–583. 10.1016/j.ejmg.2016.09.018, PMID: 27667191

[ref9] El-Sayed W. Parry D. A. Shore R. C. Ahmed M. Jafri H. Rashid Y.. (2009). Mutations in the beta propeller WDR72 cause autosomal-recessive hypomaturation amelogenesis imperfecta. Am. J. Hum. Genet. 85, 699–705. 10.1016/j.ajhg.2009.09.014, PMID: 19853237 PMC2775821

[ref10] Feske S. (2010). CRAC channelopathies. Pflugers Arch. 460, 417–435. 10.1007/s00424-009-0777-5, PMID: 20111871 PMC2885504

[ref11] Geoffroy V. Pizot C. Redin C. Piton A. Vasli N. Stoetzel C.. (2015). VaRank: a simple and powerful tool for ranking genetic variants. PeerJ 3:e796. 10.7717/peerj.796, PMID: 25780760 PMC4358652

[ref12] Huckert M. Stoetzel C. Morkmued S. Laugel-Haushalter V. Geoffroy V. Muller J.. (2015). Mutations in the latent TGF-beta binding protein 3 (LTBP3) gene cause brachyolmia with amelogenesis imperfecta. Hum. Mol. Genet. 24, 3038–3049. 10.1093/hmg/ddv053, PMID: 25669657 PMC4424950

[ref13] Jiang L. Alber J. Wang J. Du W. Yang X. Li X.. (2012). The *Candida albicans* plasma membrane protein Rch1p, a member of the vertebrate SLC10 carrier family, is a novel regulator of cytosolic Ca2+ homoeostasis. Biochem. J. 444, 497–502. 10.1042/BJ20112166, PMID: 22530691

[ref14] Laugel-Haushalter V. Langer A. Marrie J. Fraulob V. Schuhbaur B. Koch-Phillips M. (2012). From the transcription of genes involved in ectodermal dysplasias to the understanding of associated dental anomalies. Molecular Syndromology 3, 158–168. 10.1159/000342833 23239958 PMC3507273

[ref15] Nieminen P. Lukinmaa P.-L. Alapulli H. Methuen M. Suojärvi T. Kivirikko S.. (2011). DLX3 homeodomain mutations cause tricho-dento-osseous syndrome with novel phenotypes. Cells Tissues Organs 194, 49–59. 10.1159/000322561, PMID: 21252474

[ref16] Smith C. E. L. Poulter J. A. Antanaviciute A. Kirkham J. Brookes S. J. Inglehearn C. F.. (2017). Amelogenesis imperfecta; genes, proteins, and pathways. Front. Physiol. 8:435. 10.3389/fphys.2017.00435, PMID: 28694781 PMC5483479

[ref17] Vaser R. Adusumalli S. Leng S. N. Sikic M. Ng P. C. (2016). SIFT missense predictions for genomes. Nat. Protoc. 11, 1–9. 10.1038/nprot.2015.123, PMID: 26633127

[ref18] Wang S. Choi M. Richardson A. S. Reid B. M. Seymen F. Yildirim M.. (2014). STIM1 and SLC24A4 are critical for enamel maturation. J. Dent. Res. 93, 94S–100S. 10.1177/0022034514527971, PMID: 24621671 PMC4107542

[ref19] Zhao Y. Yan H. Happeck R. Peiter-Volk T. Xu H. Zhang Y.. (2016). The plasma membrane protein Rch1 is a negative regulator of cytosolic calcium homeostasis and positively regulated by the calcium/calcineurin signaling pathway in budding yeast. Eur. J. Cell Biol. 95, 164–174. 10.1016/j.ejcb.2016.01.001, PMID: 26832117

