# Supplementary material for: A New SLC10A7 Homozygous Missense Mutation Responsible for a Milder Phenotype of Skeletal Dysplasia With Amelogenesis Imperfecta
Source: Front Genet. 2019 May 28;10:504. doi: 10.3389/fgene.2019.00504 (PMC6546871; doi:10.3389/fgene.2019.00504)
Supplement: Supplementary file 1 [file Data_Sheet_1.DOCX]

**SUPPLEMENTARY MATERIAL**

**Supplementary Figures**

**Supplementary Figure 1: Slc10A7 antisense probe sequence, 918 bp.** GAACATGGTTGGTCTTAGAGGCATAGTCTACAAGGATGCCTCCTATTCACAGACTGTAAGAGCAACCGGACTTTAAATTTATTTCTACCCTTCATCTGCTGGATGACCTTGGGCAAGTCATTTTAATATCCCTAGCTGCCAGTCACAATTTATTAAATATTATAATTAAAGTACCTCAAGGTATTATTGTGAACTATTGACGGGTGTAAAGTTCTTGCAGTATAGTCTGACACAATGAACACTCTATGTCGTCACTTGTCATCATCGTCATCATTATCATCATCACCACATTGTTTGTGTATGCCAAACAGGAAGCAACCCTTTCAAATGTGGTCCATAAATATGACATCCATACACATAGTGCTTAGTGCTGTCTTCAATGGGGTTCTGTTGGCGGAAGATTCTGAGGCAGGTTGAATACAAGAAATGCATAGGGGTGAAAGGAGCAGGGAGGGGAAGCTGTTTCCCACCAAAGCCTTCCACAGCCTGTCCTAATGCCTCTCTCTGGGGCTGGGCCGACTCCTCTGAGTGGTTAGTCTTCATGTTTTCTCACAGTCATTTCAGGCTGGCCTCACTCAAACCCATAGGAAAGAAGCAGCAAACAGAGCAGGTAGATCCATACAGAACACCATAGCAGCCACCACACAGACACTCTTACAATTAACTTTAAACTTAGAAAATAGGCTAAATTTAGTGTAATTGGGGTGATTTAAAAGCAATCAAAGATTATGACTTCATTAAATACAAAATAAAATTGAAAAATACATCCCCATGCCAATTACATAATGAGGATTTTGAATAACCATTTGCCCTGCTGCCTCTGTAGCCTCTGCTCTGTTTCTCACCTGACTCACCTGGACCTTTCTCAGTCTCCGTTCATTCCTTCGGATGGAGCTCACTCCTGCCCACCTCCCACCCAGCTTATTACTCGCCTGACTCTTGAACTCACCTCTCATTTGAACGG

**Supplementary Figure 2: Energy dispersive X-ray spectrum of the dental calculus material (A) and dental enamel (B).** The palladium peak comes from the metal coating for SEM.





**Supplementary Figure 3: Sanger analysis on a *SETD9* variant.** The variant is not segregating consistently with the disease.





**Supplementary Tables**

**Supplementary Table 1: List and characteristics of *SLC10A7* primers used in this study.**

| **Primers used in this study** | | | |
| --- | --- | --- | --- |
|  | Primer name | Sequence (5' -> 3') | Position |
| RefSeq Gene NM_001300842.2 | SLC10A7-ex11F | TTTTGGTGACCCATTTCAGA | c.848-160-> c.848-141 |
|  | SLC10A7-ex11R | CCTCAACCCTCCTGTTCAAA | c.993+162-> c.993+181 |
| RefSeq Gene NM_153706.3 | SETD9-ex6F | TTCCTCATGGAAATCAAATGC | c.813-141-> c.813-121 |
|  | SETD9-ex6R | GAAATTCCTATTCCAATTAAGTCTCA | c.*125-> c.*100 |

**Supplementary Table 2: Summary of the whole exome sequencing analysis.**

| **Patient** | **I.1** | | **I.2** | | **II.4** | |
| --- | --- | --- | --- | --- | --- | --- |
| **Type of sequence variant** | SNV | indel | SNV | indel | SNV | indel |
| **Total number of variants** | 85146 | 11044 | 85766 | 11020 | 83647 | 11198 |
| **Variants with an allele frequency <1%** | 1909 | 296 | 1859 | 265 | 1736 | 241 |
| **Exclusion of 5'UTR, 3'UTR, downstream, upstream and intron locations without local splice effect prediction** | 818 | 78 | 802 | 68 | 767 | 52 |
| **Exclusion of synonymous variants without local splice effect prediction** | 625 | 74 | 600 | 60 | 577 | 50 |
| **Variants consistent with recessive transmission** | 6 compound heterozygous  and 25 homozygous variants  in 31 genes | | | | | |

After variant filtration, 25 homozygous and 6 heterozygous variants in 31 genes were left for investigation on their putative involvement in the patient’s disease.

**Supplementary Table 3: Homozygous and compound heterozygous variants after filtration.** Criteria of selection for *SlC10A7* variant and exclusion of the other putative variants.

**Supplementary Table 4: Microarray data on *Slc10a7* expression at mouse tooth cap stage.** The gene was expressed at the same level in each type of tooth at E14.5.

| **Gene symbol** | **Probe Set ID** | **Gene Description** | **mRNA Accession** | **Expression in inferior molars** | **Expression in superior molars** | **Expression in inferior incisors** | **Expression in superior incisors** |
| --- | --- | --- | --- | --- | --- | --- | --- |
| **Slc10a7** | **10572989** | **Solute carrier family 10**  **member 7** | **NM_029736** | **9,29±0,10** | **9,24±0,03** | **9,31±0,13** | **9,39±0,13** |

**Supplementary Methods**

**Whole exome sequencing**

The sequence reads were aligned to the reference sequence of the human genome (GRCh37) using the Burrows-Wheeler Aligner (BWA V7.12) (Li and Durbin, 2010). The HaplotypeCaller module of the Genome Analysis ToolKit (GATK, v.3.4.46) (DePristo et al., 2011) was used for calling both SNV and indel. Structural Variations (SV) were called using CANOES

(Backenroth et al., 2014).

**Bioinformatic analysis**

Annotation and ranking of SNV/indel were performed by VaRank (Geoffroy et al., 2015) in combination with the Alamut Batch software (Interactive Biosoftware, France). Very stringent criteria were applied to filter out non-pathogenic variants. These included (1) excluding variants represented with an allele frequency of more than 1% in public variation databases-including the 1000 Genomes (The 1000 Genomes Project Consortium et al., 2015), the gnomAD database (Exome Aggregation Consortium et al., 2016), or our internal exome database, (2) excluding variants in 5′ or 3′ UTR, (3) excluding variants with intronic locations and no prediction of local splice effect, and (4) excluding synonymous variants without pathogenic prediction of a local splice effect. Annotation of SV were performed by AnnotSV (Geoffroy et al., 2018). Variant effects on the nearest splice site were predicted using MaxEntScan (Yeo and Burge, 2004), NNSplice (Reese et al., 1997) and Splice Site Finder (Shapiro and Senapathy) programs.Very stringent criteria were applied to filter out non-pathogenic variants consitent with a recessive mode of transmission.

**Sample collection and Sanger sequencing and segregation**

Saliva samples were collected from the affected daughter and her unaffected parents and siblings using the OG-250 Oragene®DNA kit (DNA Genotek Inc., Ottawa, Ont., Canada, [www.dnagenotek.com](file:///C:\Users\virginie\AppData\Local\Temp\www.dnagenotek.com)). Genomic DNA was extracted from saliva according to standard protocols using the prepIT-L2P Oragene®DNA kit (DNA Genotek Inc.). The amplification of the specific region of interest was performed on 50 ng genomic DNA template. Primers were designed with Primer 3 (<http://frodo.wi.mit.edu/primer3>). The primer sequences are detailed in supplementary table 3. The PCR product was then purified and the bidirectional Sanger sequencing was performed by GATC Sequencing Facilities (Konstanz, Germany).

**Electron microscopy**

Immediately after extraction, teeth were washed with physiological serum and stored at 4°C in 70% ethanol. Specimens were then dehydrated in a graded series of ethanol and transferred in a propylene oxide/epon resin (v/v) solution. After embedding in Epon 812 (Euromedex, Souffelweyersheim, France), teeth were sectioned sagittally and polished with diamond pastes with decreasing granulometry down to 0.1 µm (Escil, Chassieu, France).

Polished sections were etched with 20% (w/w) citric acid, thoroughly rinsed with distilled water, and dehydrated in a graded series of ethanol solutions. The dry samples were first analyzed using an optical numeric microscope (KEYENCE, Osaka, Japon) and then assayed using the VHX-5000 Communication software. Then specimens were prepared for scanning electron microscopy and sputter-coated with a gold-palladium alloy (20/80) using a HUMMER JR sputtering device (Technics, CA, USA). The coated samples were documented using a Quanta 250 FEG scanning electron microscope (FEI Company, Eindhoven, The Netherlands) operating with an accelerating voltage of the electrons of 10 kV. Specimens also underwent chemical analysis. Energy dispersive X-ray microanalysis was performed with an EDAX Octane Plus spectrometer (AMETEK Materials Analysis Division, 91 McKee Dr, Mahwah, NJ 07430, USA).

**Supplementary References**

Backenroth, D., Homsy, J., Murillo, L. R., Glessner, J., Lin, E., Brueckner, M., et al. (2014). CANOES: detecting rare copy number variants from whole exome sequencing data. *Nucleic Acids Res.* 42, e97. doi:10.1093/nar/gku345.

DePristo, M. A., Banks, E., Poplin, R., Garimella, K. V., Maguire, J. R., Hartl, C., et al. (2011). A framework for variation discovery and genotyping using next-generation DNA sequencing data. *Nature Genetics* 43, 491–498. doi:10.1038/ng.806.

Exome Aggregation Consortium, Lek, M., Karczewski, K. J., Minikel, E. V., Samocha, K. E., Banks, E., et al. (2016). Analysis of protein-coding genetic variation in 60,706 humans. *Nature* 536, 285–291. doi:10.1038/nature19057.

Geoffroy, V., Herenger, Y., Kress, A., Stoetzel, C., Piton, A., Dollfus, H., et al. (2018). AnnotSV: An integrated tool for Structural Variations annotation. *Bioinformatics*. doi:10.1093/bioinformatics/bty304.

Geoffroy, V., Pizot, C., Redin, C., Piton, A., Vasli, N., Stoetzel, C., et al. (2015). VaRank: a simple and powerful tool for ranking genetic variants. *PeerJ* 3. doi:10.7717/peerj.796.

Li, H., and Durbin, R. (2010). Fast and accurate long-read alignment with Burrows–Wheeler transform. *Bioinformatics* 26, 589–595. doi:10.1093/bioinformatics/btp698.

Reese, M. G., Eeckman, F. H., Kulp, D., and Haussler, D. (1997). Improved splice site detection in Genie. *J. Comput. Biol.* 4, 311–323. doi:10.1089/cmb.1997.4.311.

Shapiro, M. B., and Senapathy, P. Laboratory of Statistical and Mathematical Methodology, Division of Computer Research and Technology, National Institutes of Health, Bethesda, MD 20892, USA. *Nucleic Acids Research*, 20.

The 1000 Genomes Project Consortium, Gibbs, R. A., Boerwinkle, E., Doddapaneni, H., Han, Y., Korchina, V., et al. (2015). A global reference for human genetic variation. *Nature* 526, 68–74. doi:10.1038/nature15393.

Yeo, G., and Burge, C. B. (2004). Maximum entropy modeling of short sequence motifs with applications to RNA splicing signals. *J. Comput. Biol.* 11, 377–394. doi:10.1089/1066527041410418.
